# The VDR gene FokI polymorphism is associated with gestational diabetes mellitus in Turkish women

**DOI:** 10.1186/s12881-019-0820-0

**Published:** 2019-05-16

**Authors:** Mahmut Apaydın, Selvihan Beysel, Nilnur Eyerci, Ferda Alparslan Pinarli, Mustafa Ulubay, Muhammed Kizilgul, Ozhan Ozdemir, Mustafa Caliskan, Erman Cakal

**Affiliations:** 1 Department of Endocrinology and Metabolism, Diskapi Yildirim Beyazit Teaching and Research Hospital, Ankara, Turkey; 2 Department of Medical Biology, Baskent University, Ankara, Turkey; 3 Department of Genetic Research, Diskapi Yildirim Beyazit Teaching and Research Hospital, Ankara, Turkey; 4 Department of Obstetrics and Gynecology, Gulhane School of Medicine, Ankara, Turkey; 5 Department of Obstetrics and Gynecology, Ankara Numune Teaching and Research Hospital, Ankara, Turkey

**Keywords:** VDR gene, FokI, Gestational diabetes

## Abstract

**Background:**

The association between the vitamin D receptor (*VDR*) gene and gestational diabetes mellitus (GDM) has not been investigated in Turkish pregnant women. We aimed to investigate associations between *VDR* gene *BsmI* (rs15444410), *ApaI* (rs7975232), *FokI* (rs19735810), and *TaqI* (rs731236) single nucleotide polymorphisms (SNPs) and GDM.

**Material-methods:**

This case-control study comprised 100 women with GDM and 135 pregnant women without GDM. The *VDR* polymorphism was evaluated using Sanger-based DNA sequencing.

**Result:**

*VDR* gene *ApaI*, *BsmI*, and *TaqI* SNPs did not differ between women with and without GDM (each, *p* > 0.05). *ApaI*, *BsmI*, and *TaqI* were not associated with GDM risk. The *VDR* gene *FokI* CT/TT genotype was associated with an increased GDM risk (CT vs. CC, OR = 1.84, 95% CI: [1.05–3.23], *p* = 0.031; TT vs. CC, OR = 3.95, 95% CI: [1.56–9.96], *p* = 0.002; CT/TT vs. CC, OR = 2.29, 95% CI: [1.35–3.89], p = 0.002; and CT/CC vs. TT, OR = 3.02, 95% CI: [1.23–7.38], *p* = 0.012). The *FokI-TT* genotype was more associated with younger age and higher glucose, HbA1c, and HOMA-IR than the CC and CT genotype. *FokI*-*T* was positively correlated with log-HOMA-IR (r = 0.326, *p* = 0.004). FokI SNPs were independently associated with GDM after adjusting for BMI and age (β = 1.63, 95% CI: [1. 2-4.2], p = 0.012). There were no associations between the *FokI*, *ApaI*, *BsmI* and *TaqI* haplotypes and GDM.

**Conclusion:**

*VDR* gene *FokI* SNPs were independently associated with having GDM in Turkish women. *VDR* gene *FokI* SNPs might contribute to insulin resistance of developing GDM.

## Background

Gestational diabetes mellitus (GDM) is defined as glucose intolerance diagnosed during pregnancy [1]. The prevalence of GDM shows differences among ethnic populations and ranges from 1 to 14% [2]. GDM is characterized by increased insulin resistance, hyperglycemia, and obesity [1, 3–5]. Genetic and environmental factors play an important role in the etiology of GDM [3]. Women with a family history of diabetes mellitus (DM) are at risk of GDM. Women with a history of GDM are at risk of type 2 DM (T2DM) in the future [1–5]. Genetic variations related to ß-cell dysfunction and insulin resistance have been shown to contribute to the development of GDM [1, 3, 5, 6]. The vitamin D receptor (*VDR*) gene is actively involved in the insulin metabolic pathway. Vitamin D shows its cellular activity by binding to VDR. Vitamin D plays a role in insulin secretion [7]. Vitamin D deficiency was associated with pre-eclampsia, insulin resistance, and GDM [8]. Active vitamin D shows efficacy by binding to VDR and it has a wide range of genetic variations [9]. The complex of vitamin D and its receptor is a transcription factor that plays a role in the regulation of insulin secretion from pancreatic beta cells [10]. VDR acts as a ligand-dependent transcription factor and it is a member of the nuclear hormone receptor family. The *VDR* gene is localized on chromosome 12q13.1, which consists of 11 exons [11–13]. This complex affects immune system regulation [11]. It has an effect on the proliferation, differentiation, and activation of immune cells and cytokine production, and accordingly, DM development [10–12]. Vitamin D deficiency leads to defects in insulin synthesis and secretion [10, 11, 13].

*VDR* polymorphisms have been associated with type 1 DM (T1DM) [11] and T2DM [13–15]. *BsmI* (A > G, rs1544410), *ApaI* (A > C, rs7975232), *TaqI* (T > C, rs731236), and *FokI* (C > T, rs2228570) are human VDR single-nucleotide polymorphisms (SNPs). *VDR* gene *Bsml*, *ApaI*, and *TaqI* SNPs are found in 3 prime untranslated regions where gene expression is regulated. *FokI* leads to T > C substitution at exon 2, thus the first translation initiation region is removed, and consequently transcriptional activity of VDR is changed [13, 16]. Both insulin resistance and impaired insulin secretion play a role in the pathogenesis of GDM and T2DM [1, 3, 6]. The association between *VDR* gene SNPs and GDM has been investigated in a few studies [12, 16–19]. *VDR* gene SNPs and GDM have not been investigated in Turkish pregnant women. The present study aimed to investigate associations between VDR gene SNPs (*Taq*, *BsmI*, *FokI* and *ApaI*) and GDM in Turkish pregnant women.

## Methods

### Study population

Pregnant women who were referred to the Obstetrics and Gynecology Clinic of our tertiary hospital in Ankara from 2014 to 2015 were included in this case-control study. Women with GDM (*n* = 100) and non-diabetic pregnant controls (*n* = 135) were included. The pregnant women were aged 22-38 years and the pregnancy age was 24–28 weeks. Gestational age was assessed from the date of the last menstrual period and clinical assessment. A 2-hour, 75 g oral glucose tolerance test (OGTT) at 24 to 28 weeks’ gestation was performed for all pregnant women, irrespective of family history of DM or any other risk factors for GDM. Glucose concentrations after fasting, and 1 and 2 h after glucose administration < 92 mg/dL, < 180 mg/dL, and < 153 mg/dL, respectively, were considered normal. If a patient’s glucose concentration was higher than these values, the patient was diagnosed as having GDM [2]. Women with GDM, who were diagnosed with these criteria, aged 22-38 years, and with pregnancy age 24–48 weeks were included the study. Women with GDM women with chronic disease such as hypertension, thyroid disorders, cardiac, and hepatic or renal dysfunction were excluded. Women without GDM aged 22-38 years and with a pregnancy age of 24–28 weeks who had no diabetes, hypertension, thyroid disorders, cardiac, and hepatic or renal dysfunction were included in the study as controls. Weight, height, and systolic and diastolic blood pressure (BP) were measured in all participants. Serum glucose, insulin, and glycated hemoglobin (HbA_1c_) concentrations were measured. Insulin resistance was calculated using the homeostasis model assessment-insulin resistance (HOMA-IR): [fasting plasma insulin (μIU/mL) X fasting plasma glucose (mg/dL)] / 405 [20]. The study was approved by the Diskapi Yildirim Beyazit Teaching and Research Hospital Ethics Board (Number: 26.02.2015–12/21) and written consent was obtained from all participants.

### Genotyping

Genetic analyses for *VDR* gene SNPs *FokI* (rs2228570), *BsmI* (rs1544410), *ApaI* (rs7975232), and *TaqI* (rs731236) were performed using Sanger-based DNA sequencing. Genomic DNA was isolated from collected peripheral blood samples of the subjects using a DNA Isolation Kit (Roche Diagnostics, Indianapolis, IN, USA). Genotyping of each *HNF1A* gene polymorphism was independently performed using a prevalidated fluorescence-based allele-specific polymerase chain reaction (PCR) assay, KASPar (KBiosciences, Hoddesdon, UK), which was performed on a Rotor-Gene Q real-time cycler (Qiagen, Hilden, Germany) according to the manufacturer’s instructions. Allele discrimination was made using *Rotor*-*Gene Q software* v.*2.3.1* (Qiagen, Hilden, Germany). The genotype identification was performed blind without information on clinical phenotypes.

### Statistical analysis

Statistical analysis was performed using SPSS 18.0 (SPSS, Inc) software. Variables are presented as mean ± standard deviation (SD) or median (min-max), percentages (%), odds ratios (*OR*), 95% confidence intervals (CI). Normality was tested using the Kolmogorov-Smirnov and Shapiro-Wilk *W* test. SNPs are expressed as allelic frequency (q) or prevalence of genotypes (%). Categorical variables were analyzed using the Chi-square test or Fisher’s exact test, where appropriate. Student’s *t*-test was used for comparisons of normally distributed continuous variables or log-transformed variables between the two groups. The Hardy-Weinberg equilibrium (HWE) at individual loci was assessed using the Chi-square test. Multiple logistic regression analysis and Fisher’s exact test were tested using the following models: dominant (major allele homozygotes vs. heterozygotes + minor allele homozygotes), recessive (major allele homozygotes + heterozygotes vs. minor allele homozygotes) and codominant (major allele homozygotes vs. heterozygote and minor allele homozygotes vs. major allele homozygotes). Pair-wise linkage disequilibrium (LD) and correlation coefficients (r^2^) were analyzed using the HAPLOVIEW program. We made a variable reflecting all possible combinations of *BsmI-ApaI-TaqI* genotypes for each SNP. Statistical significance was defined as a *p* < 0.05.

## Results

Obesity (46.2 vs. 18.0%, *p* = 0.001) and insulin resistance (72.4 vs. 7.2%, p = 0.001) were higher in women with GDM than in the non-GDM controls. Serum glucose, insulin, HOMA-IR, HbA1c, BMI, and BPs were higher in the GDM group than in the control group (*p* < 0.05). 25(OH)D was lower in women with GDM than in the controls (*p* < 0.05). The characteristics of the pregnant women are shown in Table 1. The four SNPs in the control group were within the HWE. Minor allele frequency and the HWE are shown in Table 2.

**Table 1:** Characteristics of subjects

	Non-GDM (*n* = 135)	GDM (*n* = 100)	*P*
Age (year)	29.13 ± 5.20	29.41 ± 5.02	0.704
Gestational age (weeks)	26.62 ± 1.48	26.04 ± 1.67	**0.014**
Height (cm)	159.01 ± 5.90	158.52 ± 4.95	0.665
Weight (kg)	68.58 ± 9.65	75.08 ± 10.07	**0.002**
BMI (kg/m^2^)	26.19 ± 4.01	29.94 ± 4.18	**0.003**
Glucose (mg/dl)	76.19 ± 8.93	105.77 ± 7.81	**0.005**
İnsulin (μIU/ml)	8.01 ± 1.69	12.83 ± 4.07	**0.002**
HOMA-IR	1.52 ± 0.48	3.36 ± 1.14	**0.003**
HbA1c (%)	4.99 ± 0.25	5.69 ± 0.38	**0.004**
25(OH)D	17.60 ± 10.24	12.04 ± 8.51	**0.001**
Systolic BP (mmHg)	101.37 ± 11.44	111.77 ± 14.31	**0.008**
Diastolic BP (mmHg)	66.01 ± 7.46	71.08 ± 7.22	**0.012**

*BMI* body mass index, *HOMA-IR* homeostasis model assessment-insulin resistance, *HbA1c* hemoglobin A1c, *BP* blood pressure, *GDM* gestational diabetes mellitus

Student’s *t* test was used for normally distributed continuous variables or log-transformed variables between two groups

Bold represents significant *p*-values

**Table 2:** Minor allele frequency and Hardy-Weinberg Equilibrium of VDR gene SNPs

	Risk allele	MAF for study sample	p for HWE in control
Apa I rs7975232	C	0.54	0.23
TaqI rs731236	C	0.35	0.78
BsmI rs15444410	G	0.38	0.15
FokI rs2228570	T	0.29	0.20

*MAF* minor allele frequency, *HWE* Hardy-Weinberg Equilibrium

The Hardy-Weinberg equilibrium (HWE) at individual loci was assessed by Chi-Square test

The distributions of the *VDR* gene SNPs are shown in Table 3. The frequency of *VDR* gene *ApaI* rs7975232, *TaqI* rs731236 and *BsmI* rs1544410 did not differ between women with and without GDM in a codominant model and dominant model and recessive model (*p* > 0.05, each). *VDR* gene *ApaI*, *TaqI*, and *BsmI* SNPs were not associated with GDM. The frequency of *VDR* gene *FokI* rs2228570 differed between women with and without GDM (*p* < 0.05). Compared with the controls, *FokI CT* genotype (CT vs. CC, OR = 1.84, 95% CI: [1.05–3.23], *p* = 0.031) and TT (TT vs. CC, OR = 3.95, 95% CI: [1.56–9.96], *p* = 0.002) genotype were associated with an increased GDM risk in a codominant model, and CT/TT carriers had increased 2.2 odds of having GDM (CT/TT vs. CC, OR = 2.29, 95% CI: [1.35–3.89], p = 0.002) in a dominant model. Compared with the controls, TT genotype carriers had increased 3.02 odds of having GDM (CT/CC vs. TT, OR = 3.02, 95% CI: [1.23–7.38], *p* = 0.012) in a recessive model. Gestational age was lower in *FokI-TT* genotype compared with CC and CT genotype (*p* < 0.05). Glucose, HbA1c, and HOMA-IR were higher in the *FokI-TT* genotype compared with the CC and CT genotypes (p < 0.05) (Table 4). *FokI-T* (risk allele) was positively correlated with log-HOMA-IR (r = 0.326, *p* = 0.004). In the logistic regression analysis, *FokI* SNPs were independently associated with GDM after adjustig for BMI and age (β = 1.63, 95% CI: [1. 2-4.2], *p* = 0.012).

**Table 3:** Genotype analysis of VDR gene SNPs

	Non-GDM (*n* = 134)	GDM (*n* = 100)	OR (95% CI)	*P*
**ApaI** rs7975232 (%)				
Co-dominant Wild type AA	19.4	17.0		
Heterozygous AC	56.7	52.0	1.04 (0.51–2.12)	0.985
Homozygous CC	23.9	31.0	1.48 (0.67–3.25)	0.326
Dominant (AA/AC + CC)			1.17 (0.59–2.30)	0.639
Recessive (AA+AC/CC)			1.43 (0.80–2.56)	0.225
**TaqI** rs731236 (%)				
Co-dominant Wild type TT	40.0	44.0		
Heterozygous CT	49.6	42.0	0.76 (0.44–1.33)	0.353
Homozygous CC	10.4	14.0	1.22 (0.52–2.84)	0.633
Dominant (TT/CT + CC)			0.84 (0.50–1.43)	0.539
Recessive (TT + CT/CC)			1.40 (0.63–3.10)	0.396
**BsmI** rs1544410 (%)				
Co-dominant Wild type AA	31.9	42.0		
Heterozygous AG	57.0	44.0	0.58 (0.33–1.02)	0.062
Homozygous GG	11.1	14.0	0.95 (0.41–2.22)	0.916
Dominant (AA/AG + GG)			0.64 (0.37–1.10)	0.109
Recessive (AA+AG/GG)			1.30 (0.59–2.83)	0.506
**FokI** rs2228570 (%)				
Co-dominant Wild type CC	60.0	41.0		
Heterozygous CT	34.1	43.0	1.84 (1.05–3.23)	**0.031**
Homozygous TT	5.9	16.0	3.95 (1.56–9.96)	**0.002**
Dominant (CC/CT + TT)			2.29 (1.35–3.89)	**0.002**
Recessive (CC + CT/TT)			3.02 (1.23–7.38)	**0.012**

*GDM* gestational diabetes mellitus

SNPs were expressed as allelic frequency (q) or prevalence of genotypes (%)

Categorical variables were analyzed with Chi-square test or Fisher’s exact test, where appropriate

Multiple logistic regression analysis and Fisher’s exact test were tested using models: dominant (major allele homozygotes vs heterozygotes + minor allele homozygotes), recessive (major allele homozygotes + heterozygotes vs minor allele homozygotes) and codominant (major allele homozygotes vs heterozygote and minor allele homozygotes vs major allele homozygotes)

Bold represents significant *p*-values

**Table 4:** Association between the VDR gene FokI SNPs and clinical features of GDM women

	CC (wild)	CT	TT	P^a^	P^b^	P^c^
				CC/CT	CC/TT	CT/TT
Age (year)	30.04 ± 5.24	29.46 ± 5.31	29.14 ± 3.79	0.515	0.315	0.588
Gestational age (weeks)	26.60 ± 1.66	26.43 ± 1.50	25.42 ± 1.39	0.893	**0.031**	**0.033**
BMI (kg/m^2^)	27.95 ± 4.11	29.16 ± 4.63	28.61 ± 3.56	0.311	0.264	0.690
Glucose (mg/dl)	87.06 ± 17.04	91.80 ± 16.66	99.42 ± 14.53	0.152	**0.002**	**0.020**
İnsulin (μIU/ml)	10.19 ± 4.01	9.88 ± 3.71	12.17 ± 3.60	0.380	0.036	**0.009**
HOMA-IR	2.30 ± 1.31	2.33 ± 1.16	3.05 ± 1.20	0.888	**0.009**	**0.013**
HbA1c (%)	5.29 ± 0.50	5.33 ± 0.47	5.47 ± 0.33	0.738	**0.011**	**0.019**

^a^CC genotype vs CT genotype ^b^ CC genotype vs TT genotype

^c^CT genotype vs TT genotype

*BMI* body mass index, *HOMA-IR* homeostasis model assessment-insulin resistance, *HbA1c* hemoglobin A1c

Student’s *t* test was used for normally distributed continuous variables or log-transformed variables between two groups

Bold represents significant *p*-values

## Discussion

This case-control study showed that *VDR* gene *FokI* SNPs were independently associated with having GDM in Turkish women. The frequency of the *VDR* gene *FokI* TT and CT genotype was increased in women with GDM compared with the non-GDM controls. The frequency of *VDR* gene *ApaI*, *BsmI*, and *TaqI* SNPs did not differ between women with and without GDM with no association. *VDR FokI* SNPs might contribute to insulin resistance in the development of GDM.

Our results showed that 25(OH)D concentrations were lower in the GDM group than in the control group. Vitamin D deficiency was associated with insulin resistance and GDM [8]. The *VDR* gene has a role in the metabolic pathway of insulin [9]. *VDR* gene variations have beenshown to be correlated in the development, progression, and complications of T2DM [13–15]. The present study showed that *VDR* gene *FokI* SNPs were independently associated with an increased risk of GDM in Turkish women (β = 1.63, 95% CI: [1. 2-4.2], *p* = 0.012). Our study suggested that *VDR* gene *FokI* SNPs might be associated with having GDM. We found that the frequency of *VDR* gene *ApaI*, *TaqI*, and *BsmI* did not differ between women with and without GDM. *VDR* gene *ApaI*, *TaqI*, and *BsmI* SNPs were not associated with GDM. The *VDR* gene *FokI* SNP showed significant differences between women with and without GDM. *VDR* gene *FokI* (variant or heterozygotes) compared to wild-type (CC) SNP revealed a significant association. *VDR* gene *FokI* rs2228570 TT (TT vs. CC, OR = 3.95, 95% CI: [1.56–9.96], *p* = 0.002) and CT heterozygotes (CT vs. CC, OR = 1.84, 95% CI: [1.05–3.239, *p* = 0.031) were associated with having GDM, compared with the controls. *VDR* gene *FokI* SNPs might contribute to developing GDM in the Turkish population.

Similar to our results, *FokI* homozygous SNPs were reported as prevalent in patients with DM and GDM [12, 13]. Aslani et al. reported that *VDR* gene *FokI* SNPs were associated with GDM in an Iranian population [12]. Another study reported that *ApaI* and *Taq* SNPs were associated with GDM in an Iranian population [16]. These results are incompatible with our study, thus we showed that *ApaI*, *Taq*, and *BsmI* SNPs were not associated with GDM. *BsmI* and *FokI* SNPs were not associated with GDM in a Saudi Arabian population [17]. Vural and Maltas et al. showed that *TaqI* SNPs were not associated with T2DM in a Turkish population [15]. Dilmec et al. reported that *TaqI* SNPs were associated with T2DM, but *ApaI* and *FokI* SNPs were not associated with T2DM in a Turkish population [14]. Previous studies investigating the *VDR* gene in Turkish patients with T2DM were compatible with our study. Hence, we supposed that *Taq* and *ApaI* were not associated with having T2DM in the Turkish population.

*VDR* gene *Taq*, *BsmI* or *ApaI* SNPs were not associated with diabetic microvascular complications but only *FokI* SNPs were associated with diabetic neuropathy in a Caucasian population [13]. Meta-analysis reported that only *FokI* SNPs were found as a risk factor for T2DM. *Taq*, *BsmI* or *ApaI* SNPs were not associated with DM [21]. These reports were similar to the present study; we showed that only *FokI* SNPs were associated with having GDM. A meta-analysis showed that only BsmI SNPs were associated with autoimmune T1DM in an Asian population [11]. We supposed that autoimmunity might contribute to the association between *BsmI* SNPS and having T1DM. The inconsistency between studies might result from ethnic diversity and environmental factors on *VDR* variations in different populations [12].

The present study showed that the *FokI-T* (risk allele) was positively correlated with log-HOMA-IR. Assessment of allele frequency distribution showed a significant association of the *FokI* variant allele (T) on susceptibility toward to GDM. We supposed that the *FokI* variant might contribute to impaired insulin resistance and metabolic disorder in developing GDM. Hence, *FokI* SNPs might have a role in the pathogenesis of GDM.

*BsmI*, *ApaI*, and *TaqI* polymorphisms of the *VDR* gene are found in the three-primer untranslated region (3′-UTR) and have been shown to be in strong linkage disequilibrium (LD) [21]. The *FokI* polymorphism was reported as an independent marker of the *VDR* gene because it has not been shown to be in linkage disequilibrium with any of other *VDR* polymorphisms [12]. Our study reported that *VDR* gene *FokI, ApaI, BsmI* and *TaqI* haplotypes were not associated with GDM, and *ApaI, BsmI* and *TaqI* polymorphisms were not shown in LD. *ApaI* and *BsmI* polymorphisms of the *VDR* gene, both in intron 8, are considered as silent SNPs. These polymorphisms do not change the amino acid sequence of the encoded protein, but they might affect gene expression by modulating stability of mRNA [21]. The *TaqI* polymorphism is located at codon 352 in exon 9 of the *VDR* gene. The *TaqI* TT genotype (absence of restriction site) is related to lower active vitamin D3 [21]. The only locus with impact on the structure of VDR protein is the *FokI* polymorphism, which is located on the 5′ end region of the *VDR* gene. The *VDR* gene *FokI* polymorphism is functional because it is found in a coding sequence. The *FokI* polymorphism is located in the first ATG starting code of VDR protein. *FokI* is involved in thymine to cytosine (T/C) substitution at exon 2, the first translation initiation region is removed, and transcriptional activity of VDR is changed [12, 13, 16, 22]. It alters the ACG codon, which is found ten base pairs upstream from the translation starting codon and leads to the generation of an additional starting codon. Two different VDR isoforms occur with transition of allele T to C in ATG. When initiating translation starts from this alternative site in the thymine variant, it generates a longer VDR protein comprised of 427 amino acids. The gene is transcribed in normal length if there is a restriction site. Thus, the C/C allele codes a 424-amino acid protein and the T/T allele codes a 42 7-amino acid protein. The longer VDR protein has low activity in transcription, accordingly activation is decreased in target cells [12, 13]. The *FokI* T/T genotype, *FokI* C/C, showed 1.7-fold greater function in vitamin D-dependent transcriptional activation of a reporter through the regulation of a vitamin D response element [22]. The *FokI* rs2228570 polymorphism is the only *VDR* gene polymorphism involved in the generation of altered protein expression [12]. Apart from obesity and insulin resistance, complex genetic (ethnicity) and non-genetic (epigenetic) mechanisms may have a role in the etiology of GDM [9].

The cross-sectional design, small sample size, and absence of postpartum follow-up are the limitations of this study.

## Conclusion

This study showed that *VDR* gene *FokI* SNPs were independently associated with an increased risk of GDM in Turkish pregnant women. *VDR* gene *FokI* SNPs may be considered as a risk factor for metabolic disorders in GDM. *VDR FokI* SNPs may have a role in the etiology of GDM. Further studies in different populations are needed to confirm these results.

## Abbreviations

25(OH) vitamin D_3_: 25-hydroxyvitamin D_3_
BMI: body mass index
BP: blood pressure
GDM: gestational diabetes mellitus
HbA1c: hemoglobin A1c
HOMA-IR: homeostasis model assessment-insulin resistance index
LD: Pair-wise linkage disequilibrium
